# Welfare assessment of novel on-farm killing methods for poultry

**DOI:** 10.1371/journal.pone.0212872

**Published:** 2019-02-22

**Authors:** Jessica E. Martin, Victoria Sandilands, Julian Sparrey, Laurence Baker, Laura M. Dixon, Dorothy E. F. McKeegan

**Affiliations:** 1 The Royal (Dick) School of Veterinary Studies and The Roslin Institute, Easter Bush Campus, The University of Edinburgh, Edinburgh, United Kingdom; 2 Institute of Biodiversity, Animal Health and Comparative Medicine, College of Medical, Veterinary & Life Sciences, University of Glasgow, Glasgow, United Kingdom; 3 Animal and Veterinary Science Research Group, Scotland’s Rural College (SRUC), Edinburgh, United Kingdom; 4 Livetec Systems Ltd, Silsoe, Bedford, United Kingdom; Tokat Gaziosmanpasa University, TURKEY

## Abstract

There is a need for novel mechanical devices for dispatching poultry on farm following the introduction of EU Regulation (EC) no. 1099/2009 On the Protection of Animals at the Time of Killing. We examined three novel mechanical killing devices: Modified Armadillo, Modified Rabbit Zinger, a novel mechanical cervical dislocation device; and traditional manual cervical dislocation. The four killing methods were tested on 230 chickens across four batches. We measured behavioural, electroencephalogram and post-mortem outcomes in anesthetized laying hens and broilers at two life stages (juveniles and adults/slaughter age). Graeco Latin-Square designs systematically randomized killing treatment, bird type, age and kill order. All birds were lightly anaesthetized immediately prior to the killing treatment with inhalation of Sevoflurane. The novel mechanical cervical dislocation method had the highest kill success rate (single application attempt only, with no signs of recovery) of a mechanical method (96%). The Modified Armadillo was the least reliable with 49% kill success. Spectral analysis of electroencephalogram signals at 2 s intervals for successfully killed birds only revealed progressive decreases in median frequency alongside increases in total power. Later, total power decreased as the birds exhibited isoelectric electroencephalogram signal. Latencies to pre-defined spectral ranges associated with unconsciousness showed that birds subjected to manual and novel mechanical cervical dislocation achieved these states sooner than birds subjected to the modified Armadillo. Nevertheless all methods exhibited short latencies (<4 s). The Modified Rabbit Zinger had the shortest duration of reflex persistence for nictitating membrane, pupillary and rhythmic breathing post method application. Of the methods tested, the novel mechanical cervical dislocation device is the most promising mechanical method for killing poultry on-farm based on a range of behavioural, electroencephalogram and anatomical parameters. This device has the potential to fulfil the current need for a mechanical alternative to manual cervical dislocation.

## Introduction

Poultry need to be dispatched on-farm for multiple reasons (e.g. injury, sickness and for stock management), either on a group/large scale (e.g. whole-house or containerized gassing [1–2]) or as individuals (e.g. manual cervical dislocation or captive bolt [3–5]).The industry standard for killing individual chickens on-farm is cervical dislocation as it is perceived to be humane by users, and is easy to learn and perform [6]. There are two types of cervical dislocation: manual and mechanical. Both are designed to separate the skull from the vertebral column (ideally C0–C1 vertebral dislocation) and sever the spinal cord and/or brainstem and the main blood vessels supplying the brain [7–8]. Mechanical cervical dislocation is differentiated from manual dislocation by the use of an aid or tool in order to complete the action (e.g. killing cone [7]) [9]. Mechanical methods have had limited uptake within the poultry industry due to practical limitations (e.g. non-mobile killing cone). Optimally, cervical dislocation should have a concussive effect through brain stem trauma and cause death by cerebral ischemia [3,10]. Previous work on cervical dislocation (mechanical only) suggested that birds may be conscious for a significant period post-application [3,11]. Accordingly, current EU legislation, Regulation (EC) no. 1099/2009 On the Protection of Animals at the Time of Killing [9], restricts the use of manual cervical dislocation in terms of number of birds which can be dispatcher per person per day (to 70), as well as applying weight limits of individual birds killed for both manual (<3 kg) and mechanical (<5 kg) cervical dislocation methods. This creates a need to develop new mechanical devices to provide alternative methods to kill individual birds on farm which are humane and practical, as well as comply with legislation. Some new methods have been primarily developed to dispatch larger birds (e.g. CASH Poultry Killer, Turkey Euthanasia Device) [3,12–13]. However, none have been enthusiastically adopted by the poultry industry, especially for chickens (laying hens and broilers) who represent the greatest demand in terms of bird numbers.

Determination of time to loss of consciousness is crucial when ascertaining the welfare impact of a killing method. EEG (electroencephalogram) analysis is a useful tool which has been applied for this purpose in various livestock species [14–15]. The EEG represents the recording of the electrical activity of the brain through the summation of the inhibitory or excitatory postsynaptic potentials from pyramidal cells near each recording electrode. Recording electrodes can be either surgically implanted on to the surface of the dura (an electrocortigram (ECoG) or intracranial electroencephalography (iEEG)) or by resting electrodes on the scalp (an electroencephalogram (EEG) [16–18]). Analysis of EEG recordings can differentiate between different brain activity states (sleep and unconsciousness) and can identify brain death [13,19].

In the field, it is not practical to record EEG in each animal to confirm unconsciousness and death [3], therefore the absence of reflexes (e.g. pupillary, nictitating membrane) are used to determine brain death [14,20–21] and loss of consciousness (e.g. jaw tone) [3,19]. Correlation between the loss of certain reflexes and changes in EEG characteristics are not well documented in poultry, but a recent study demonstrated that the loss of jaw tone was indicative of an unconscious state in layer hens and turkeys, when the state was induced through anesthesia with sevoflurane [19]. In addition, latency to loss of posture was strongly correlated with iEEG spectral parameters associated with unconsciousness in broilers undergoing Low Atmospheric Pressure Stunning [22]. Sandercock et al [19] also showed that loss of the nictitating membrane reflex was a conservative indicator of death in layer hens and turkeys. In behaviorally confirmed unconscious states, the iEEG signal shows a sharp increase in total spectral power (PTOT), which is associated with a decrease in the median frequency (F50) and the spectral edge frequency (F95) [19,22].

The aim of this experiment was to evaluate the efficacy and welfare impact of three novel killing methods for on-farm killing of poultry using both iEEG and reflex measurements. The methods tested were the modified Armadillo (MARM), a puntilla-style device; modified Rabbit Zinger (MZIN), a penetrating captive bolt; and a novel mechanical cervical dislocation glove (NMCD), which aids the manual technique. These three killing treatments had previously been trialed with cadavers [8], where they were shown to produce sufficient anatomical trauma in order to result in death, as well as performing in the intended way. However, because the devices were novel, for ethical reasons the work was carried out in anaesthetized birds. This experiment was designed to provide results to inform the decision of whether the devices should be taken forward for further evaluation in conscious birds in comparison with manual cervical dislocation, thereby determining their potential as humane on-farm killing methods for poultry. The effects of each device were determined in three ways: (1) analysis of electrical brain activity (via iEEG recordings); (2) duration of reflexes and behaviors post killing treatment application; and (3) post mortem evaluation.

## Materials and methods

This work was performed under Home Office (UK) authority via Project and Personal Licenses and underwent review and approval by Scotland’s Rural College’s (SRUC) Animal Welfare and Ethical Review Body (AU AE 34–2012).

### Subjects and husbandry

A total of 232 female laying hens and broiler chickens, each at two different ages, were used for the study (Table 1). Graeco Latin-Square designs were used to systematically randomize killing treatment, bird type and age and kill order. The design was then split into four batches in order to accommodate surgery requirements, with all bird type x age combinations equally represented across each batch (Batch 1 = 40 birds (i.e. n = 10 per type/age) and Batches 2–4 = 64 birds per batch (i.e. n = 16 per bird type/age)). The birds were housed for two weeks prior to the experiment to allow acclimatization, and (for a subset of birds) for iEEG surgery and recovery. Birds were housed in separate rooms per bird type and age group to provide recommended commercial environmental controls. All birds were kept in floor pens with *ad libitum* food and water, wood-shavings litter and kept at lower than commercial stocking densities (layer pullet = 2.3 kg/m^2^; layer hen = 4.7 kg/m^2^; broiler chick = 1.9 kg/m^2^; and slaughter-age broiler = 3.8 kg/m^2^) and with suitable environmental enrichment (e.g. layer birds provided with nest boxes and pecking boards; all birds provided with shiny CDs suspended on string and perches).

**Table 1:** Mean (±SE) age and weight at time of killing for each bird type and age group, as well as numbers of birds allocated to each killing treatment (Modified Armadillo (MARM); Modified Zinger (MZIN); novel mechanical cervical dislocation (NMCD) and manual cervical dislocation (MCD).

Bird group	Total N	Bird age (days)	Bird weight (kg)	MARM	MZIN	NMCD	MCD
Layer pullets (Hy-Line)	64	79.1±2.1	0.89±0.02	10 (10)	10 (3)	22 (12)	22 (12)
Layer hens (Hy-Line)	64	502.6±2.2	1.76±0.03	10 (10)	10 (2)	22 (12)	22 (12)
Broiler chicks (Ross 308)	40	7.1±0.6	1.02±0.04	10 (0)	10 (0)	10 (0)	10 (0)
Slaughter-age broiler (Ross 308)	64	41.2±0.7	2.51±0.06	10 (10)	10 (2)	22 (12)	22 (12)

Within this, the number of birds that were iEEG implanted is reported in “()”.

Broiler chicks, which were not implanted with iEEG electrodes due to their small skull size, were housed in one pen as a group (L 1.5 m x W 2.5 m x H 1.5 m). Slaughter-age broilers, layer pullets and layer hens were kept singly (L 1.5 m x W 0.5 m x H 1.5m; visual and auditory contact with others) following iEEG electrode implantation surgery. Following the completion of batch 1, it became apparent that the presence of the iEEG electrode was significantly hampering the performance of one of the killing methods, the modified Zinger, and subsequently the birds in the batches assigned to the Zinger treatment (batches 2–4) did not undergo iEEG implantation surgery.

Prior to the predetermined killing days across all four batches, two birds were euthanised on welfare grounds, reducing the total N for two treatments by one bird each (MARM = 39 birds; NMCD = 75 birds).

### iEEG electrode implantation

**Fig 1:**
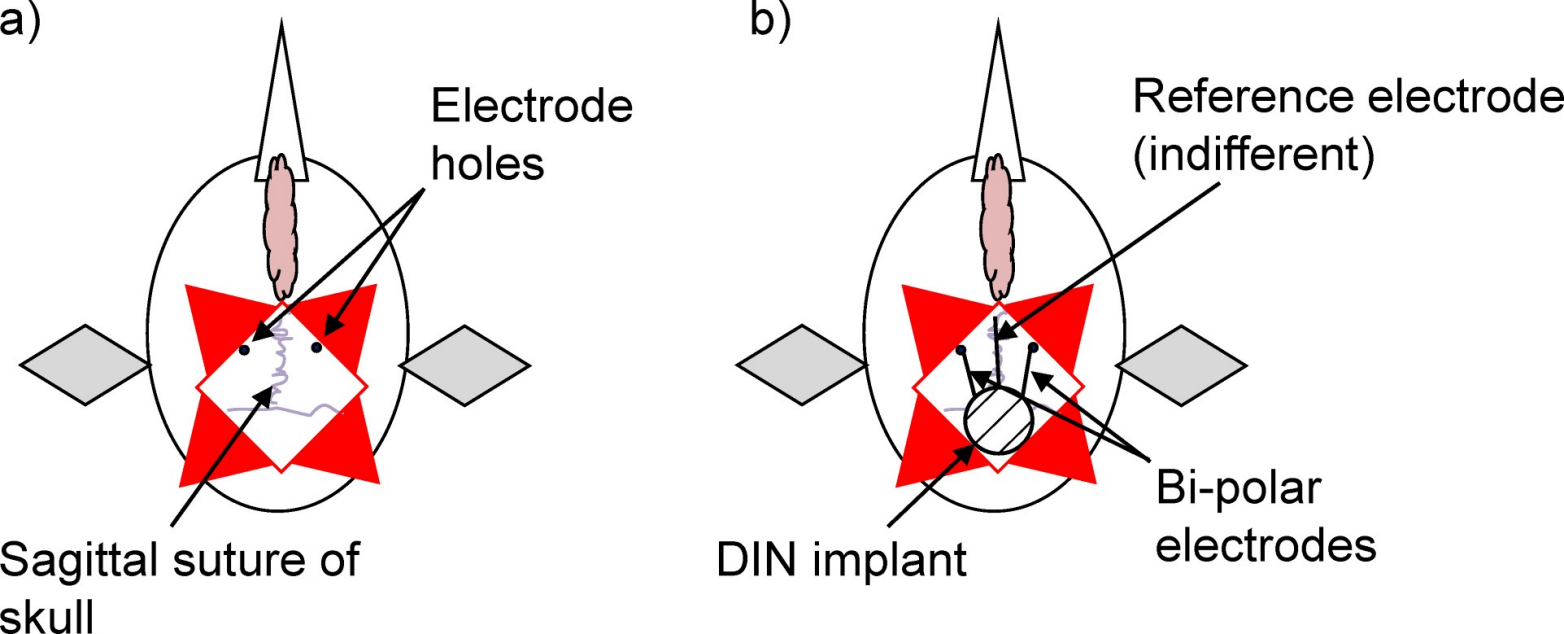
Diagrammatic representation of the location of two drill sites either side of the sagittal suture into the skull over the forebrain (a); and placement of bi-polar electrode wires (through the two drill sites) to rest, one on each of the dorsal surfaces of the right and left telencephalon and reference electrode placed between the skin and the skull (b).

Only layer pullets, laying hens and slaughter-age broilers were implanted with iEEG electrodes. Five days prior to the designated individual bird killing date across the four batches, a total of 109 birds underwent surgery to implant iEEG electrodes under general anesthesia, induced and maintained with sevoflurane (SevoFlo, Animal Health, Hampshire, UK). All birds received a pre-medication of dexmedetomidine (40 μg/kg, administered IM; Dexdomitor, Elanco, Animal Health, Hampshire, UK), approximately 30 minutes prior to surgery. At the start of surgery, carprofen (4 mg/kg, administered SC; Rimadyl, Zoestis UK Ltd, London, UK) analgesic was administered to provide post-operative pain relief. The bipolar iEEG implantation approach has been described previously [19,22]. Briefly, the iEEG signal was recorded by two 0.35 mm diameter Teflon insulated silver electrodes (World Precision Instruments Ltd., Hertfordshire, UK) connected to a socket (DIN, RS components Ltd., Corby, UK), placed on the dura through small holes drilled in the skull, one on each of the dorsal surfaces of the right and left telencephalon at their approximate rostro-caudal and medio-lateral midpoints (Fig 1). An indifferent electrode was placed between the skull and the overlying tissue under the comb. The iEEG implant was secured to the skull with dental cement (Duralay, Dental Directory Ltd., Witham, UK) and the surrounding skin was closed with sutures. After recovery from the anesthetic, birds were individually housed and closely monitored.

### Modified and novel killing devices

All killing methods applied are discussed in detail in Martin et al [8] and were designed to comply with the current European legislation, EC 1099/2009 [9]. The modified devices had been previously tested on 80 cadavers [8].

The Armadillo is a brain-stem penetration device designed to dispatch game birds in the field [6,8]. The device consists of a scissor-type mechanism (L = 17 cm); the bird’s head is placed into the ‘cup’ of the lower arm (beak facing downwards) and when ready to apply the operator squeezes the handles together, which pushes the top arm wielding the penetrating spike downwards into the back of the bird’s skull, severing the top of the spinal cord (or brain stem), and causing death by cerebral ischemia. Modifications (with the permission of the inventor) consisted of replacing the lower arm of the device in order to increase the upper (33–37 mm) and lower (19–27 mm) diameters of the metal cup and addition of insert-able padded cups to facilitate its use in a wide range of poultry [8], and the device was renamed MARM.

The Rabbit Zinger [8] is a penetrating captive-bolt device originally designed to kill rabbits. It uses the stored energy in rubber tubes to drive a penetrating bolt into the animal’s head, causing death by extensive irreversible brain damage [5,8]. The blue Power Tubes [8] were used, which require 177 N to pull the bolt into the cocked position [5–6,8]. When fired the bolt (0.6 mm diameter) delivers approximately 11.87 J of kinetic energy. The modifications, with permission of the original inventor, have been described previously [5,8]. Briefly, they consisted of the addition of three aluminum appendages added to the base of the device in order to provide a method of gently restraining the bird’s head. Also, leather washers were added to the bolt, in order to reduce the penetration depth from 3.5 to 2.5 cm. The modified version was renamed MZIN.

The novel manual cervical dislocation device (NMCD) was designed to create a mechanical method which mirrored the technique of the manual cervical dislocation method for poultry [5,8]. Briefly, the device consists of a thin supportive glove (SHOWA 370 Multipurpose Stable Glove, UK) and a moveable metal insert. The metal insert comprises two metal finger supports that are designed to fit around the bird’s head to create a secure grip, and to move independently from side-to-side in order to allow adjustment for different sizes of birds. The rounded shape of the metal fingers is designed to aid the twisting motion (performed during manual cervical dislocation [5–6]) required to dislocate the bird’s neck by enhancing the ‘rolling action’ of the hand. The blunt edge between the two metal fingers (protruding < 1 mm from the fleshy area of skin between the index and middle fingers) provides a hard edge to force between the back of the bird’s head and the top of the neck, designed to focalize the force into the desired area (i.e. a dislocation at C0–C1) when the method is applied.

Traditional manual cervical dislocation was included in the experiment as a control. This was performed by an experienced operator following the HSA’s guidelines [7].

### Experimental procedure

Across the four batches, Graeco Latin-Square designs were used to systematically randomize killing treatment, bird type and age and kill order. Killing treatment was allocated to individual birds so as not to confound killing treatment with pens. Birds were killed over four days for each batch, with 10–18 birds killed per day. All birds were brought to the experiment room (separate to their housing environment) in individual pet carriers. Immediately before each killing treatment application, iEEG implanted birds were fitted with instrumentation: a reusable custom-made Lycra harness was secured using Velcro fastenings behind the bird's head and incorporated a pocket positioned on the bird's back which contained a custom-made telemetry/logging device, capable of logging simultaneous iEEG and ECG signals and described elsewhere [18,22–23]. Briefly, the logger has two signal conditioning amplifier channels, which can be used as required for amplifying electrocardiogram (ECG) and electroencephalogram (iEEG) signals [23]. Each signal chain starts with a differential input stage using low-voltage operational amplifier devices (OPA2344, Burr Brown) in a standard ‘instrumentation amplifier’ configuration. This is followed by an integrator/high-pass filter stage, which removes the effects of dc common-mode potentials at the input without suffering the long post-transient recovery times associated with typical ac coupled designs. A variable gain stage follows that is based on a resistor network switched by a quad analogue switch device (MAX292, Maxim). The signal from the final gain stage signal is fed into the 12-bit analogue-to-digital converter (ADC) on the microcontroller. There are 16 gain settings and setting 1 was used for our recordings. The default units we apply at import to Spike are microvolts, and as such the absolute voltages at each electrode are not defined in the raw data. However, this is not vital given the nature of both subjective and power spectrum analysis which focuses on patterning and frequency of the signal.

Two ‘physiological waveform’ input channels were used to record ECG and iEEG (sampling rate 1000 Hz). Logging was triggered and stopped with an external switch and logged data were recorded onto industry-standard ‘micro-SD’ memory cards (SanDisk 32GB, Maplin Electronics Ltd. Rotherham, UK). Two identical loggers were alternated. The logger harness was additionally secured to the birds with elastic bandage (Vetrap, 3M, Elanco, Animal Health, Hampshire, UK). All birds were lightly anaesthetized immediately prior to the killing treatment being applied via a “fast knockdown” method using a face mask (induction via inhalation of sevoflurane (SevoFlo, Animal Health, Hampshire, UK), at an 8% concentration vaporized in 100% oxygen for a maximum of 20 seconds). Birds were confirmed as non-responsive to pain immediately prior to killing treatment application by applying a sharp toe and comb pinch. The approach used represented a balance between ensuring that birds were unconscious when killed but minimizing ongoing effects of anesthesia on the iEEG after killing. Light anesthesia as induced by this method is transient, and birds readily recover (within approximately 4 s, demonstrated in pilot work), so if not killed the birds would have rapidly regained consciousness. All killing methods were applied by a single fully trained operator, holding a valid Welfare of Animals at the Time of Killing (WATOK) license for killing operations outside a slaughterhouse.

Kill success was defined as only one application attempt with no signs of recovery (e.g. return of rhythmic breathing). If any signs of recovery continued for 15 s (i.e. 1 interval measure) the bird was emergency euthanised; the method of euthanasia was killing treatment dependent in order to prevent post mortem examination data being voided (e.g. for manual cervical dislocation and NMCD it was application of the CASH Poultry Killer .22 (CPK 200–1 grain (65 mg) gunpowder cartridge); for MZIN and MARM it was by manual cervical dislocation.

### Behavioral observations

Seven reflexes and behavior (Table 2) were assessed as present or absent at 15 s intervals post killing treatment application, until a consecutive 30 s absence of all behaviors and reflexes was observed. All of these reflexes and behaviors have been validated in previous research as indicators of either brain death or unconsciousness [3,5,12,14,19]. Assessment of the presence and absence of the behaviors and reflexes was conducted by two observers (JM and LB): observer 1 (JM) assessed reflexes and behaviors associated with the bird’s head, while observer 2 (LB) assessed measures relating to the body and limbs of the bird. Head and body measures were recorded simultaneously by both observers, but in a specific order within each observer (i.e. head measures were always measured in the order of jaw tone, nictitating membrane and pupillary reflex; while body measures were recorded in the order of rhythmic breathing, wing flapping, leg paddling and cloacal movement). Binary sampling methods were used, meaning that if a reflex/behaviour was present during any point of a 15 s interval it was defined as present for the entire interval [24], providing a maximal measure of reflex/behaviour durations post killing treatment to therefore infer a conservative measure. Data were reported as the mean of the maximum durations. If a reflex or behaviour could not be recorded (e.g. pupillary reflex was concealed due to damage to the eye) the data was recorded as missing.

**Table 2:** List of behaviors recorded post-killing treatment, as well as the procedure used to assess them as present or absent.

Reflex/ Behavior	Procedure
Pupillary (light) reflex	Constriction of the pupil when light is directed into the eye from a medical pen light approximately 5 cm from the corneal surface.
Nictitating membrane reflex	The nictitating membrane (palpebra tertia) transiently closes over the surface of the eye when the medial canthus is touched with a probe.
Rhythmic breathing	Observations of >3 consecutive breaths from visual confirmation of the rib cage moving up and down rhythmically.
Jaw/mandible tone	Muscle resistance observed in response to downward manipulation and pressure applied to the lower beak.
Cloacal movement	Visual observation of sporadic opening and closing of the cloaca in a “puckering” movement.
Wing flapping	Observation of clonic flapping of the wings in a sporadic fashion.
Leg paddling	Observation of clonic movement of the legs in a sporadic fashion.

### iEEG analysis

The logged data files were uploaded into a data acquisition and analysis program (Spike 2 Version 4.2, Cambridge Electronic Design, Cambridge, UK). iEEG activity was sampled continuously in 2 s epochs during a two minute baseline period (awake bird), during “fast knockdown” with anesthetic (15–20 s), at killing method application and post-kill activity until all behaviors and reflexes had ceased for a minimum of 30 s. Visual inspection was used to eliminate obvious movement artefacts which rendered the signal meaningless, while epochs that were apparently affected by minor electrical noise interference were subject to post hoc ‘filtering’ using the data interpolation technique [22]. The iEEG was analyzed by producing a power spectrum of each epoch using a Fast Fourier Transform algorithm (1024, Hanning window, resolution 0.976 Hz bins), with each epoch displayed and analyzed with a band-pass filtered between 0.5 – 100Hz [19,22], and unfiltered raw iEEG data were saved.

Three epochs (midpoint ± 10 s either side) of iEEG wave activity were obtained and analyzed in the 2-min baseline period (conscious bird). One epoch was analyzed during the “fast knockdown” period. Overlapping 2 s epochs were obtained from -2 s to +5 s (i.e. -2 to 0, -1 to +1, 0 to +2, +1 to +3, +2 to +4, and +3 to +5 s) relative to the time of killing treatment application (estimated kill application time = 0 s). From +5 s to +59 s, a continuous series of non-overlapping 2 s epochs were analyzed. Thereafter 2 s epochs were sampled from the midpoint every 15 s, until three consecutive samples were judged to be isoelectric.

Three spectral variables were calculated with coded Genstat (Genstat, 14^th^ Edition, Rothamsted Research, UK) programs: total power (PTOT), defined as the total area under the power spectrum curve [19];median frequency (F50), the frequency below which 50% of the iEEG power resides [25] and the spectral edge frequency (F95), the frequency below which 95% of the iEEG power resides [25].

Epochs within the first 2 s post-killing were removed (accounting for an additional 2 s required to position the bird and remove its head from the mask) from the analysis for calculating latencies to unconsciousness ranges (F50 ≤ 12.7 Hz and ≤ 6.8 Hz) [19,22]. Therefore, in order to minimize the effects of the anesthetic.

### Post-mortem evaluations

Post-mortem examination was carried out immediately after confirmation of death. Specific post-mortem measures were recorded for each killing treatment as their target areas were different. For all killing treatments binary yes/no measures were recorded for broken skin, external blood loss and subcutaneous hematoma. For the MZIN and MARM, seven specific measures were recorded: skull penetration location; binary measures of damage to the left forebrain, right forebrain, cerebellum, midbrain and brainstem; and the presence/absence of an internal brain cavity hematoma. For killing treatments which caused trauma to the neck of the bird, seven specific post-mortem measures were assessed: four binary measures were recorded for dislocation of the neck, vertebra damage (e.g. intra-vertebra dislocation/break), damage to neck muscle, and whether the spinal cord was severed. The level of cervical dislocation was recorded (e.g. between C0-C1, C1-C2, C2-C3, etc.), as well as the gap length (cm) between the dislocated cervical vertebra. The number of carotid arteries severed was also recorded as either zero, one or both.

Device success was defined as when the device caused the expected anatomical damage, as well as producing sufficient damage which would be expected to result in rapid death. Device success criteria were device specific: MARM–back of skull was penetrated and brain stem severed; MZIN–skull was penetrated and severe damage to a minimum of one gross area of the brain; for both manual cervical dislocation and NMCD–complete dislocation at C0-C1, with severance of the spinal cord and both carotid arteries, no breakage of the skin or sign of crushing injury to the trachea or esophagus.

### Statistical analysis

All data collected at the bird level were summarized in Microsoft Excel (2010) spreadsheets and analyzed using Genstat (14^th^ Edition). Statistical significance was termed by a threshold of 5% probability based on F tests. A *p* value ranging from >0.05 - <0.10 was defined as a statistical trend. Summary graphs and statistics were produced at the bird level. Statistical comparisons for kill success and device success were conducted via Generalized Linear Mixed Models (GLMMs), using the logit link function and binomial distribution.

Post-mortem measures were divided into neck damage methods (i.e. NMCD and manual cervical dislocation) and head damage methods (MZIN and MARM) and analyzed separately. Statistical comparisons were performed on sub-sets of data to remove failure birds (i.e. kill success “no”) in order to prevent data skewing. All post-mortem binary measures (e.g. skin break yes/no) and categorized measures (e.g. brain damage grade) were analyzed via GLMMs using logit link function and binomial distribution. Device success was used as a fixed effect within all the models.

For the reflex/behavior durations, statistical comparisons were performed on successfully killed birds only, in order to prevent data skewing. The presence/absence of each reflex and behaviour was summarized into interval counts (e.g. present in 0–15 s = 1 count), therefore summarizing the data into means of the maximum interval counts at the bird level for each reflex, which were then converted back into the time dimension (s). GLMMs with logit link function and Poisson distributed errors were fitted to the interval counts. Overall statistical comparisons across the killing treatments were conducted. Further analysis involved sub-setting the data into two groups: (1) NMCD and MCD; and (2) MZIN and MARM, which allowed post-mortem effects to be fitted into the models as factors. Device success was used as a fixed effect within all the models.

For all models the random effects included the batch and the kill day. All fixed effects were treated as factors and classed as categorical classifications and all interactions between factors were included in maximal models.

## Results

### Kill and device success

A total of 33 birds were not killed at the first attempt across the killing treatments: MARM = 19/39 birds; MZIN = 30/40 birds; NMCD = 72/75 birds; and MCD = 0/76 birds. The 33 birds were immediately killed with an emergency method, thus invalidating their reflex/behaviour, iEEG, ECG and post-mortem data. Kill success (defined as only one application attempt with no signs of recovery) was therefore affected by killing treatment (*F*_*3*,*229*_ = 24.46_,_ *p* < 0.001) with the MCD being the most successful method, (100.0% kill success), followed by NMCD (96.0%), MZIN (75.0%) and MARM (48.7%). Bird type, age, iEEG implantation status (whether bird had an iEEG electrode array surgically implanted), body weight, kill order and all interactions did not affect kill success. Device success (defined as when the device caused the expected anatomical damage) was significantly affected by killing treatment (*F*_*3*,*229*_ = 4.38_,_ *p* = 0.004), with the MZIN being the most successful (75.0%), matching its kill success. The NMCD, MCD and MARM all had less than 45% device success. Device success was also affected by bird age (*F*_*6*,*229*_ = 4.48_,_ *p* = 0.034), being more likely to be achieved in both younger birds (69.9 ± 0.1%) compared to the two groups of older birds (58.9 ± 0.1%). There was no effect of bird weight, bird type, iEEG implantation status or kill order on device success.

### iEEG responses

**Fig 2:**
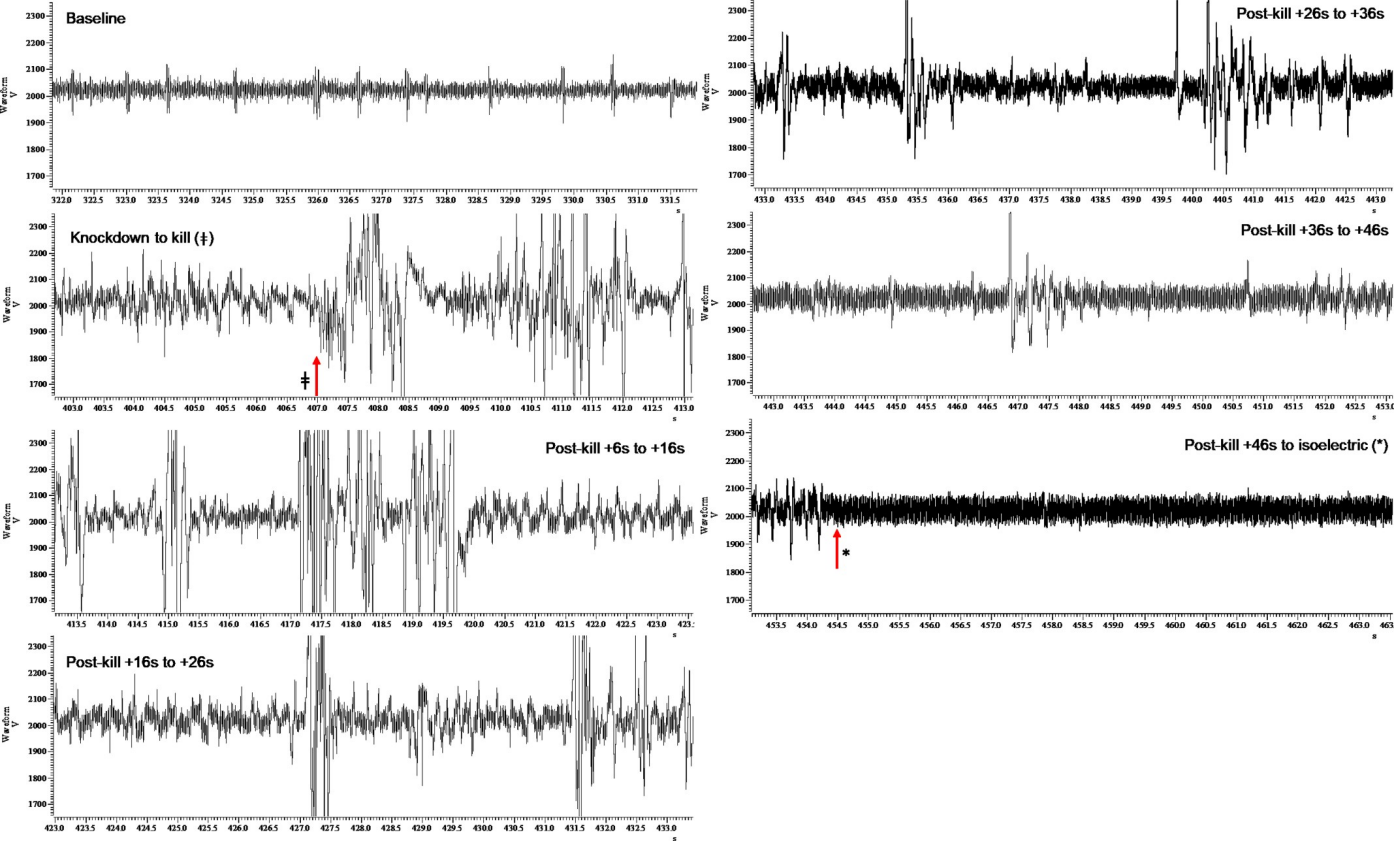
A representative series of iEEG trace excerpts (each 10 s duration, data from Broiler 44) illustrating the typical appearance of the iEEG for manual cervical dislocation (MCD). Time points are labelled for killing method application (ǂ) and transition into permanent isoelectric (*). The y-axis units are microvolts, and the x-axis units are seconds.

**Fig 3:**
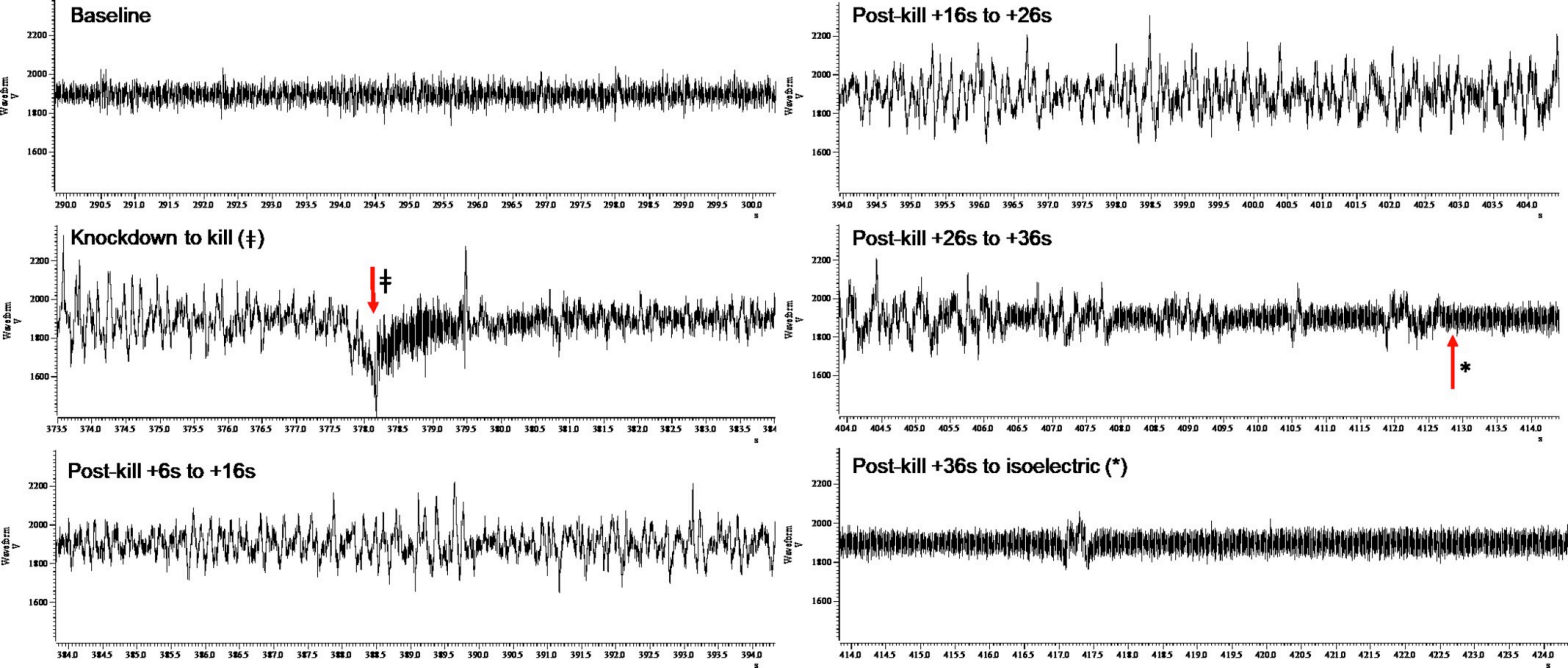
A representative series of iEEG trace excerpts (each 10 s duration, data from Layer pullet 44) illustrating the typical appearance of the iEEG for novel mechanical cervical dislocation (NMCD). Time points are labelled for killing method application (ǂ) and transition into permanent isoelectric (*). The y-axis units are microvolts, and the x-axis units are seconds.

**Fig 4:**
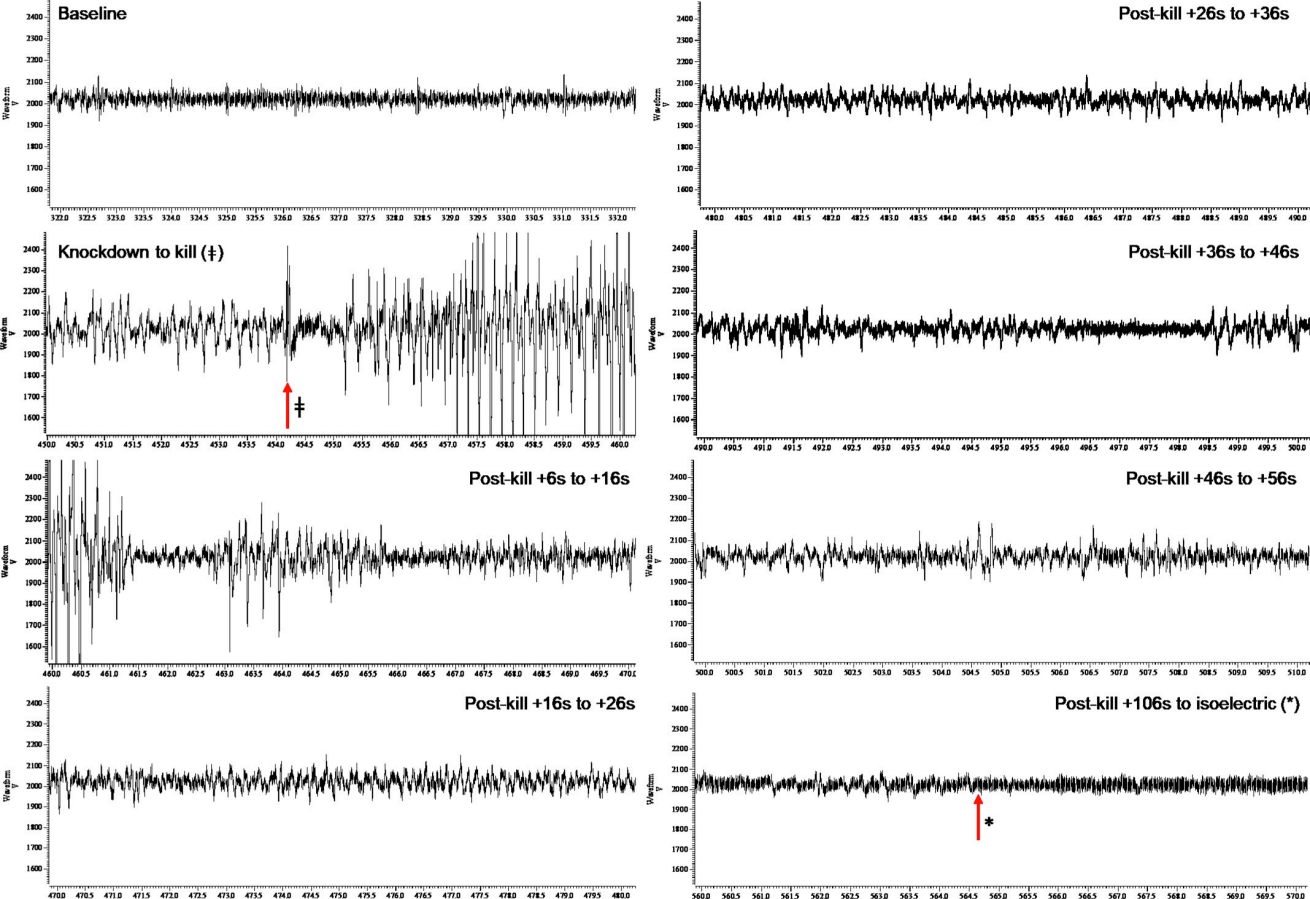
A representative series of iEEG trace excerpts (each 10 s duration, data from Layer pullet 44) illustrating the typical appearance of the iEEG for modified Armadillo (MARM). Time points are labelled for killing method application (ǂ) and transition into permanent isoelectric (*). The y-axis units are microvolts, and the x-axis units are seconds.

In total, complete or partial usable iEEG traces from baseline to knockdown and through killing to isoelectric iEEG were obtained from 74 implanted birds. Of these 74 birds, 58 birds were successfully killed and these were not evenly spread across the three killing treatments, bird types and ages. Figs 2–4 display representative series of iEEG trace excerpts (each 10 s duration) illustrating the typical appearance of the iEEG for each killing method.

In the baseline period (awake/conscious) the spectral variables of PTOT (mean = 709.6 ± 87.3 μV^2^), F50 (mean = 23.0 ± 1.3 Hz) and F95 (mean = 70.7 ± 1.5 Hz),were not significantly different between killing treatments. There were also no significant differences between killing treatments for PTOT (mean = 4001.3 ± 1016.6 μV^2^), F50 (mean = 10.2 ± 0.5 Hz) and F95 (mean = 24.2 ± 2.2 Hz) during the knock-down (unconscious/light anesthetic) phase before killing methods were applied.

Killing treatment affected the latency to F50 < 12.7 Hz (Table 3). The MCD had the shortest mean latency to F50 < 12.7 Hz, compared to NMCD and MARM, which were not different to each other. When the devices performed optimally, this significantly (*F*_*1*,*45*_ = 8.66_,_ *p* = 0.003*)* reduced the time to F50 < 12.7 Hz (device success means: yes = 1.5 ± 0.4 s; no = 5.6 ± 1.7 s). Bird type *(F*_*1*,*45*_ = 3.88_,_ *p* = 0.049*)* also had an effect, with layer hens (mean = 5.3 ± 1.6 s) exhibiting longer F50 < 12.7 Hz timings than slaughter-age broilers (means = 2.4 ± 0.7 s). The remaining fixed effects (e.g. bird weight, bird age) were not significant.

**Table 3:** Intracranial electroencephalogram (iEEG) summary statistics (mean, standard error (SE), minimum, maximum and N) of latency times for unconsciousness and isoelectric thresholds for all successful kills.

Latency	Killing treatment	Time post-kill (s)				N	*F*	*p*
		Mean	SE	Min	Max			
First time to F50 < 12.7 Hz	MCD	2.6	1.5	1	32	17	3.83	0.022
	MARM	3.5	2.6	1	20	11		
	NMCD	3.1	1.6	1	11	16		
First time to F50 < 6.8 Hz	MCD	3.2	0.3	1	32	19	4.24	0.022
	MARM	3.5	0.3	1	40	11		
	NMCD	3.1	0.3	1	16	15		
First time to isoelectric	MCD	41.8	6.3	11	80	12	23.64	<0.001
	MARM	72.0	16.1	20	170	9		
	NMCD	46.3	6.0	8	85	14		
Last time not isoelectric	MCD	39.2	5.2	2	65	13	6.20	0.002
	MARM	43.9	8.1	4	95	10		
	NMCD	21.5	5.2	10	46	15		
Non-recovery of F50 < 12.7 Hz	MCD	14.2	0.7	1	32	14	3.61	0.028
	MARM	15.1	2.1	1	58	9		
	NMCD	6.1	0.6	1	20	11		
Non-recovery of F50 < 6.8 Hz	MCD	49.0	0.9	38	58	8	5.88	<0.001
	MARM	51.1	0.7	42	58	9		
	NMCD	27.6	1.5	4	42	9		

Generalized linear mixed model results of statistical differences reported (*F* statistic and *p* value) dependent on killing treatment (Modified Armadillo (MARM); novel mechanical cervical dislocation (NMCD) and manual cervical dislocation (MCD). Number of epochs varies as not every measure was available for every bird.

Killing treatment also had an effect on the first time to F50 < 6.8 Hz, with the MARM having longer latencies to F50 < 6.8 Hz compared to NMCD and the MCD (*F*_*2*,*35*_ = 4.24_,_ *p* = 0.022*)*, which were not significantly different. Mean latency to F50 < 6.8 Hz was significantly shorter when the device application was optimal (device success means: yes = 2.3 ± 1.4 s; and no = 4.3 ± 2.2 s (*F*_*2*,*45*_ = 8.75_,_ *p* = 0.005*)*). Bird type/age also had a significant effect (*F*_*1*,*45*_ = 8.75_,_ *p* = 0.011*)* with shorter latencies for slaughter-age broilers (mean = 4.7 ± 2.1 s) compared to layer type birds (hens and pullets) (mean = 5.7 ± 1.5 s).

The first time to isoelectric and last time not isoelectric were calculated in order to provide an estimate of when brain death occurred. Killing treatment had an effect on latencies to both first (*F*_*2*,*44*_ = 23.64_,_ *p* < 0.001) and last (*F*_*2*,*38*_ = 6.20_,_ *p* = 0.002) isoelectric EEG occurrence, with the MARM having the longest latencies compared to the MCD and NMCD.

**Fig 5:**
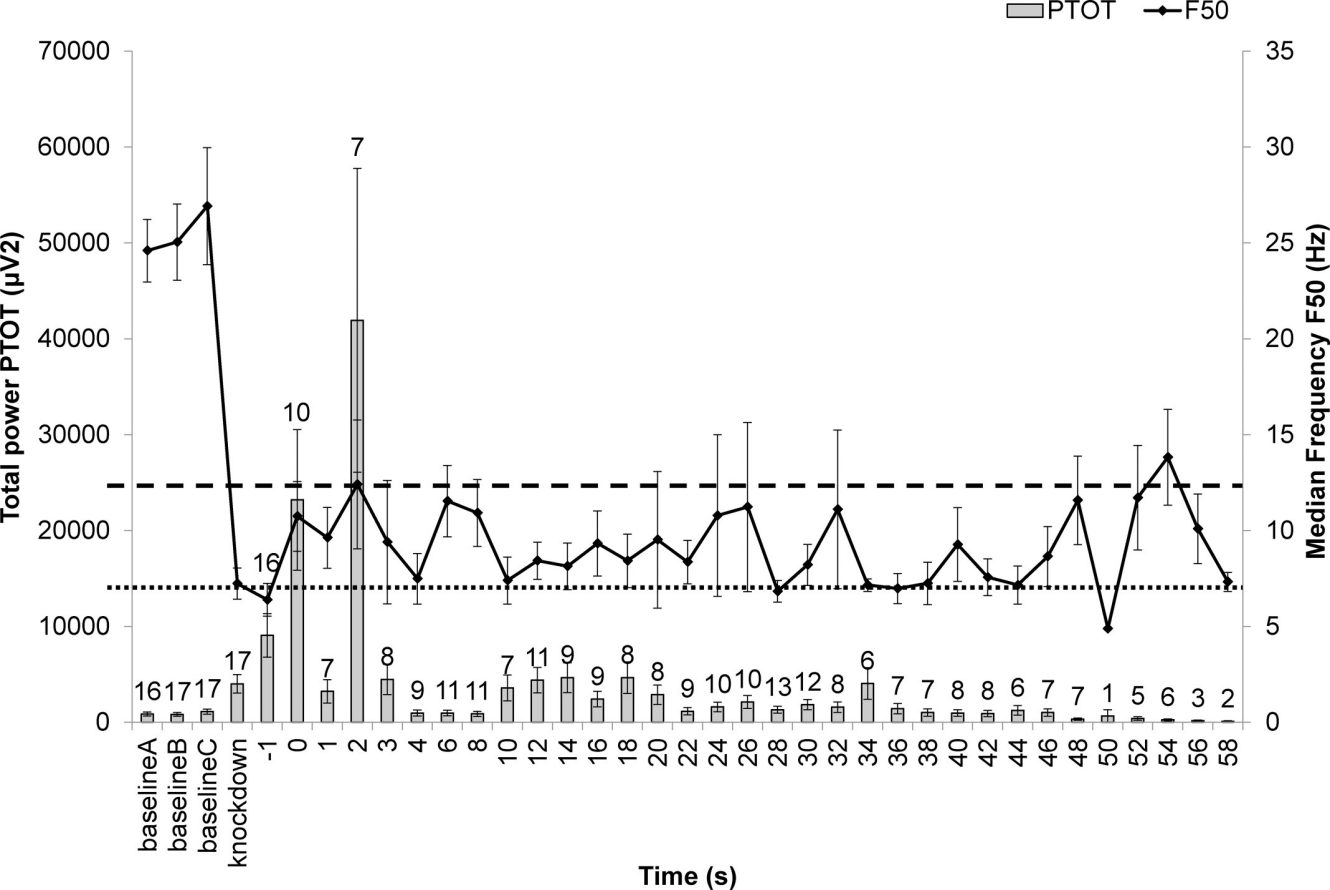
Time series of mean (±SE) PTOT and F50 spectral variables from baseline, to knock-down (anaesthetized), “kill” (application of killing method at 0 s), and every 2 s post-application for 1 minute for MCD (manual cervical dislocation). Number of epochs per time interval identified by the values above the bars (maximum N = 19)). The horizontal lines correspond to spectral unconsciousness ranges: dashed = non-responsive (F50 < 12.7 Hz); dotted = general anesthetic (F50< 6.8 Hz). Once birds were identified as brain dead they were removed from the time series, resulting in a gradual reduction in epochs at these later time points.

**Fig 6:**
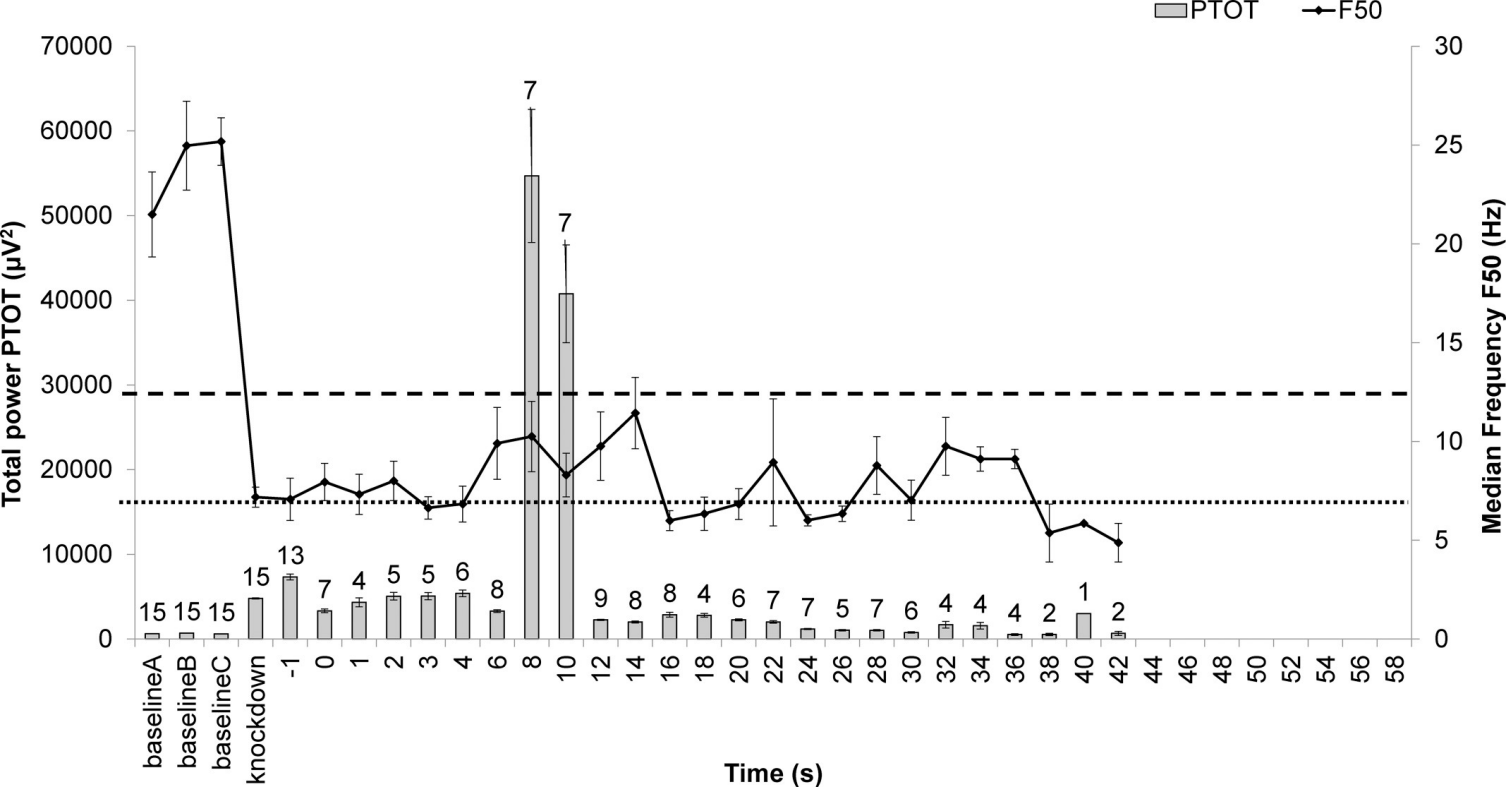
Time series for of mean (±SE) PTOT and F50 spectral variables from baseline, to knock-down (anaesthetized), “kill” (application of killing method at 0 s), and every 2 s post-application for 1 minute for NMCD (novel mechanical cervical dislocation). Number of epochs per time interval identified by the values above the bars (maximum N = 17)). The horizontal lines correspond to spectral unconsciousness ranges: dashed = non-responsive (F50 < 12.7 Hz); dotted = general anesthetic (F50< 6.8 Hz). Once birds were identified as brain dead they were removed from the time series, resulting in a gradual reduction in epochs at these later time points.

**Fig 7:**
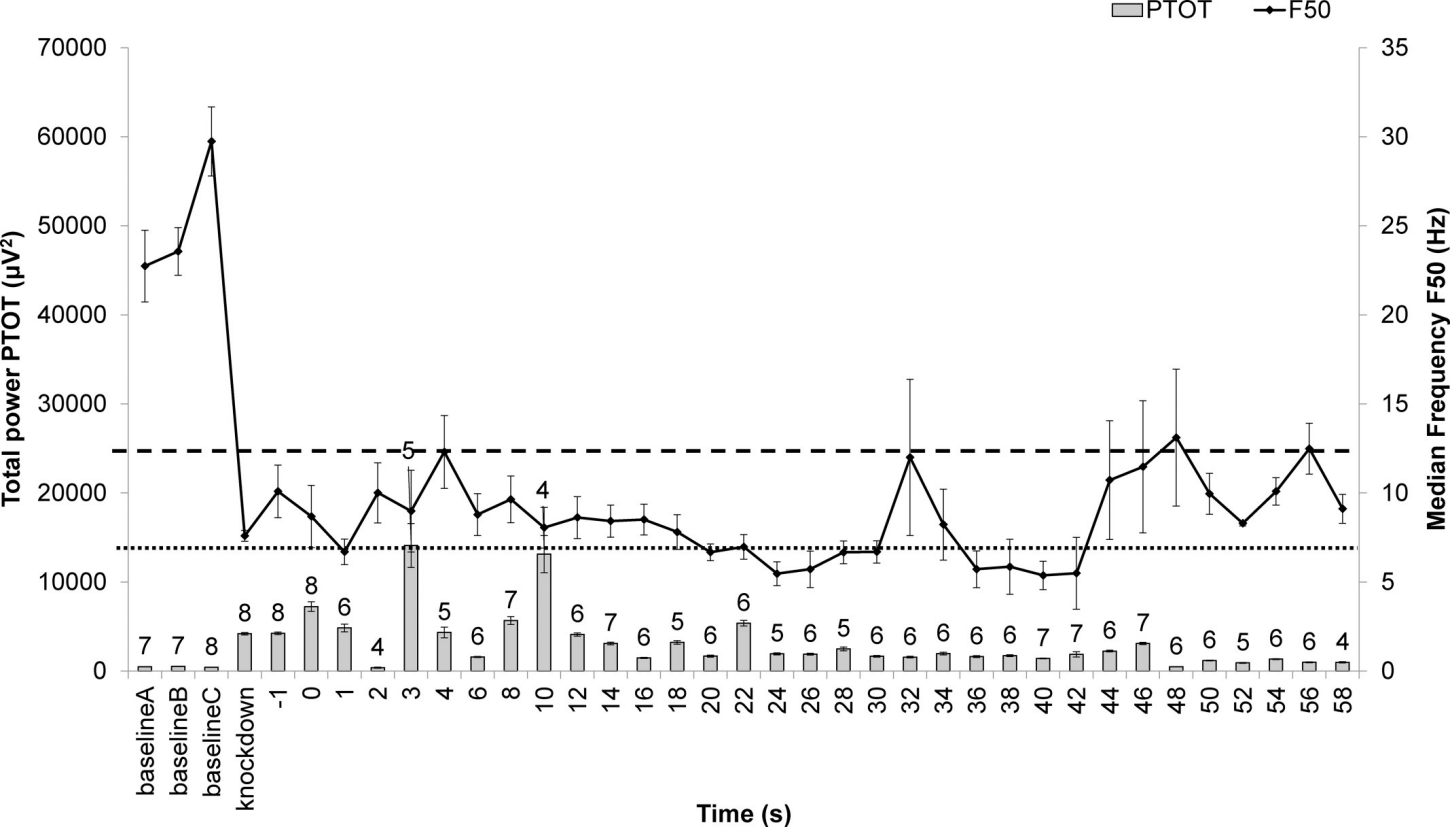
Time series for of mean (±SE) PTOT and F50 spectral variables from baseline, to knock-down (anaesthetized), “kill” (application of killing method at 0 s), and every 2 s post-application for 1 minute for MARM (modified Armadillo). Number of epochs per time interval identified by the values above the bars (maximum N = 9)). The horizontal lines correspond to spectral unconsciousness ranges: dashed = non-responsive (F50 < 12.7 Hz); dotted = general anesthetic (F50< 6.8 Hz). Once birds were identified as brain dead they were removed from the time series, resulting in a gradual reduction in epochs at these later time points.

Continuous plotting of F50 and PTOT was carried out to characterise the induction of unconsciousness in the birds and its maintenance until brain death. Both cervical dislocation killing treatments caused a sharp increase in PTOT after application (Figs 5 and 6), although the timing of the peak and magnitude were method-specific. This sharp increase was not observed in birds killed by the MARM (Fig 7). Birds in the MCD treatment remained non-responsive (means and SE below non-responsive threshold (F50 ≤ 12.7 Hz)) from the point of application for 65.6% of time intervals (21/32 intervals) and 48.2% remained within the unconsciousness threshold (F50 ≤ 6.8 Hz). An isoelectric EEG was seen in 63.2% of birds within 1 minute post method application (12/19 birds). In the NMCD treatment the majority (95.7% of time intervals) of birds remained below the non-responsive threshold (F50 ≤ 12.7 Hz) and 44.2% of time intervals within the unconsciousness threshold (F50 ≤ 6.8 Hz) post application until brain death, which all birds reached by the 42 s interval. For the MARM treatment the majority birds remained below the non-responsive threshold (F50 ≤ 12.7 Hz) from the point of application for 81.3% of time intervals (26/32 intervals). However, only 45.5% of birds reached isoelectric within 1 minute post method application (5/11 birds) and less than 40% of time intervals remained within the unconsciousness threshold (F50 ≤ 6.8 Hz) until death. Latencies to non-recovery of unconsciousness thresholds (i.e. the time point at which thresholds were never exceeded) were affected by killing treatment, with NMCD showing the shortest latencies in both cases (Table 3) compared to MARM and MCD.

### Behavioral responses

**Fig 8:**
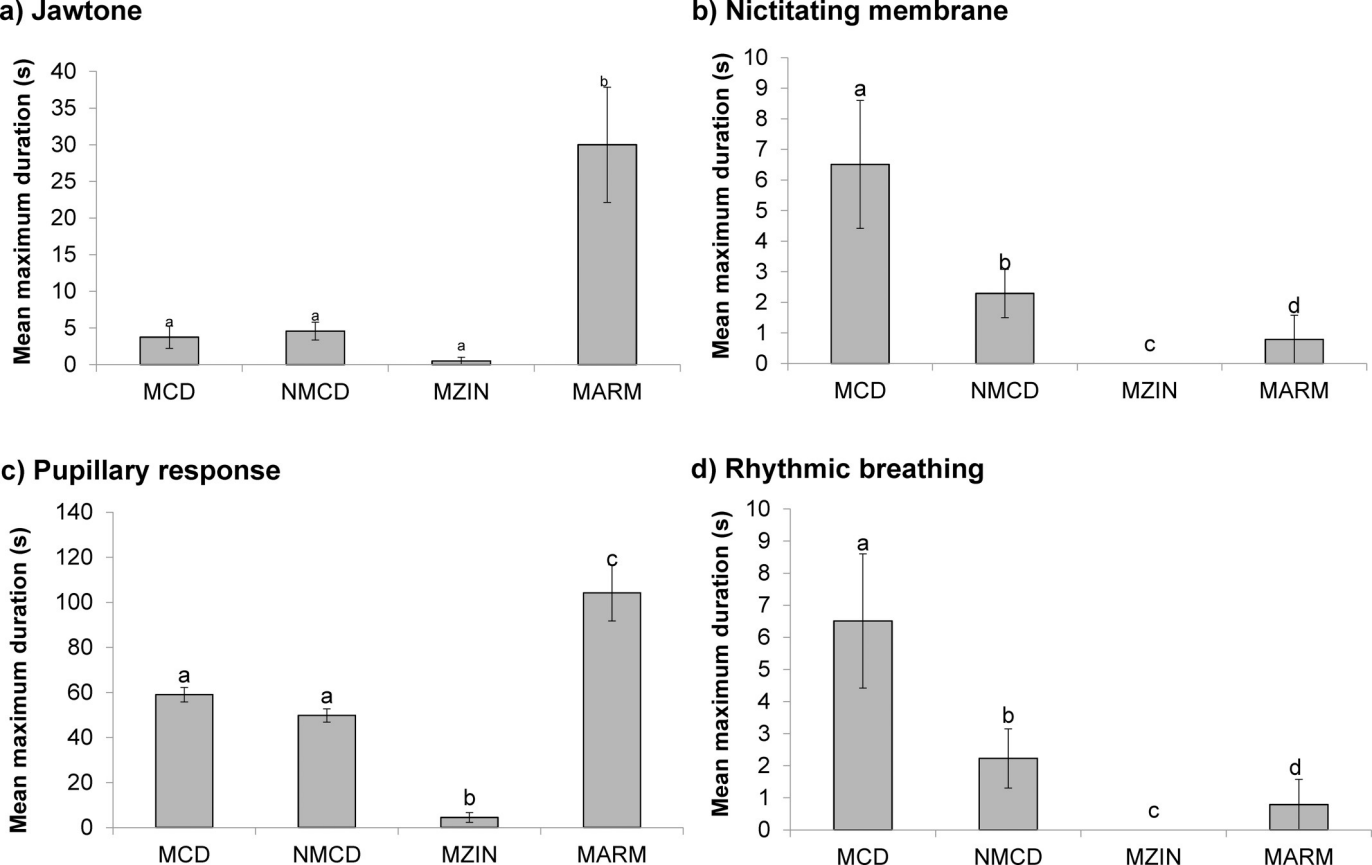
Summaries of the mean maximum durations (±SE) (s) across the three kill treatments (Modified Armadillo (MARM); novel mechanical cervical dislocation (NMCD); Modified Zinger (MZIN) and manual cervical dislocation (MCD)) for the cranial reflexes: (a) jaw tone; (b) nictitating membrane; (c) pupillary reflex; and (d) rhythmic breathing. No common superscript indicates that there is a significant difference between the treatment groups.

Killing treatment had a significant effect on observed durations of jaw tone (*F*_*3*,*195*_ = 21.11_,_ *p* <0.001); nictitating membrane reflex (*F*_*3*,*195*_ = 2.91_,_ *p* = 0.036); pupillary reflex (*F*_*3*,*195*_ = 59.50_,_ *p* < 0.001) and rhythmic breathing (*F*_*3*,*195*_ = 2.94_,_ *p* = 0.036) (Fig 8), with the (successfully killed) MZIN treated birds having the shortest durations. Table 4 shows the behavioral parameters for which there were no differences between killing treatments. Across all birds, 77.6% never showed jaw tone following application of the killing treatments. Bird weights were positively correlated with nictitating membrane reflex (*r* = 0.201, *p* = 0.005) and rhythmic breathing durations (*r* = 0.201, *p* = 0.006), with heavier birds displaying longer durations of both reflexes. Bird type had an effect on the pupillary reflex (broilers = 46.6 ± 10.5 s; layers = 72.8 ± 14.5 s, *p* < 0.001), wing flapping (broilers = 110.6 ± 5.0 s; layers = 152.4 ± 4.5 s, *p* < 0.001), leg paddling (broilers = 114.1 ± 5.4 s; layers = 155.4 ± 4.4 s, *p* < 0.001) and cloacal movement (broilers = 119.8 ± 5.5 s; layers = 159.9 ± 5.0 s, *p* < 0.001) durations post method application, with broilers exhibiting significantly shorter durations.

**Table 4:** Summary statistics (mean, SE, minimum, and maximum) of behavioural/reflex durations post killing treatment application, as well as statistical differences (*F* statistic and *p* value) dependent on killing treatment (Modified Armadillo (MARM); novel mechanical cervical dislocation (NMCD); Modified Zinger (MZIN) and manual cervical dislocation (MCD)).

Behaviour/reflex	Killing treatment	Time post-kill duration (s)				*F*	*p*
		Mean	SE	Min	Max		
Wing flapping	MCD	134.8	6.5	45.0	255.0	0.63	0.595
	NMCD	129.2	5.8	45.0	240.0		
	MZIN	138.0	7.2	60.0	225.0		
	MARM	144.5	12.6	45.0	255.0		
Leg paddling	MCD	137.8	6.5	45.0	255.0	0.94	0.424
	NMCD	131.7	6.1	45.0	240.0		
	MZIN	141.0	7.1	45.0	225.0		
	MARM	151.6	12.6	60.0	255.0		
Cloacal movement	MCD	145.7	6.5	45.0	255.0	0.37	0.778
	NMCD	140.0	6.5	45.0	255.0		
	MZIN	146.5	8.5	60.0	240.0		
	MARM	134.2	15.8	45.0	255.0		

### Post-mortem evaluations

For successfully killed birds from cervical dislocation methods, all had their necks fully dislocated and their spinal cord severed, with no intra-vertebrae damage, irrespective of cervical dislocation method. The location of the dislocation point did not differ between the two methods (*F*_*1*,*152*_ = 0.05_,_ *p* = 0.816), with the majority receiving a C0-C1 dislocation (MCD = 80.2%; NMCD = 79.2%). MCD resulted in the lowest dislocation level recorded (C4-C5), which occurred in two birds, a layer hen and a slaughter-age broiler. Age at killing (*F*_*1*,*152*_ = 10.18_,_ *p* = 0.002) and neck gap (*F*_*1*,*152*_ = 11.61_,_ *p* < 0.001) affected dislocation level, with older birds being more likely to have a lower dislocation than younger birds. Neck gap was affected by killing treatment (*F*_*1*,*152*_ = 5.59_,_ *p* = 0.022), with NMCD (mean = 6.6 ± 0.3 cm) resulting in larger neck gap sizes compared to the MCD (mean = 5.3 ± 0.3 cm). Bird type (*F*_*1*,*152*_ = 8.92_,_ *p* = 0.004) and bird age (*F*_*1*,*152*_ = 13.92_,_ *p* < 0.001) also had an effect with layers and younger birds having larger neck gap sizes compared to broilers and adults (bird type: layers = 5.9 ± 0.3 cm; broiler = 5.5 ± 0.3 cm; bird age: adult = 5.4 ± 0.3 cm; juvenile = 6.1 ± 0.2 cm). With NMCD, 23.6% of birds exhibited breakage/tearing of the neck skin, compared to 13.2% of MCD birds, although this difference was not significant. For both methods, almost all birds sustained a subcutaneous haematoma (MCD = 100.0%; NMCD = 98.6%) and severe muscle damage and tearing to the muscle in the neck (MCD = 100.0%; NMCD = 98.6%). Most birds also had one or both carotid arteries severed (MCD = 72.4%; NMCD = 87.5%), with NMCD more likely to sever one or both arteries than the MCD (*F*_*1*,*152*_ = 11.05_,_ *p* < 0.001). Larger neck gaps were more likely to result in one or both carotid arteries being severed (carotid arteries severed: zero = 3.5 ± 0.3 cm; one = 5.2 ± 0.4 cm; two = 6.6 ± 0.4 cm, (*F*_*1*,*152*_ = 32.19_,_ *p* < 0.001)).

Kill success had a significant effect on a number of post-mortem measures for both brain-trauma killing treatments (Table 5). Damage was more likely to occur when the kill was successful. Following successful kills, all birds exhibited damage/breakage to the skin, external bleeding and damage to the skull, irrespective of killing treatment. Kill success was affected by the specific location of the skull damage (*F*_*1*,*48*_ = 5.66_,_ *p* = 0.016) and killing treatment (*F*_*1*,*48*_ = 7.10_,_ *p* < 0.001). For successfully killed birds, the range of skull areas damaged was lower in both devices compared to unsuccessfully killed birds. When the MARM was successful, skull damage occurred only around the rear central area of the skull (i.e. occipital bone and foramen magnum), however, when unsuccessful the areas of damage were more varied. When successful, the MZIN damaged the middle and rear areas of the skull, again, when unsuccessful the areas of damage varied. For both devices, in some unsuccessful kills, no damage to the skull was observed, with only soft tissue damage around the head and neck (MARM = 15%; MZIN = 20%).

**Table 5:** Percentage of birds for which the post-mortem measure was present, according to killing treatment (Modified Zinger (MZIN) and Modified Armadillo (MARM)) and whether the kill was successful or not, as well as statistical differences (*F* statistic and *p* value) dependent on killing treatment.

Post mortem measure	Percentage of birds observed for kill success (Yes/No)				*F*	*p*
	MZIN		MARM			
	Yes	No	Yes	No		
Skin broken	100.0	90.0	100.0	95.0	1.17	0.088
External bleeding	100.0	80.0	100.0	90.0	1.21	0.062
Subcutaneous hematoma	90.0	100.0	89.5	85.0	1.23	0.074
Skull damage	100.0	80.0	100.0	85.0	1.19	0.124
Brain cavity hematoma	100.0	60.0	94.7	50.0	3.22	0.032
Left forebrain damage	83.3	10.0	0.0	5.0	28.8	<0.001
Right forebrain damage	83.3	0.0	0.0	10.0	22.36	<0.001
Cerebellum damage	86.7	10.0	63.2	20.0	0.83	0.737
Midbrain damage	96.7	10.0	10.5	5.0	21.48	<0.001
Brain stem damage	3.5	0.0	84.2	0.0	35.55	<0.001

In successfully killed birds, the brain damage occurred in the cerebellum and brain stem after application of the MARM, while for the MZIN all brain regions except the brain stem were subject to damage. Damage to all brain regions except for the cerebellum was affected by treatment. Bird weight, irrespective of kill method, affected whether or not the right forebrain (N = 1.67 ± 0.17 kg; Y = 1.45 ± 0.12 kg, *F*_*1*,*48*_ = 5.50_,_ *p = 0*.*022*) and brain stem (N = 1.45 ± 0.10 kg; Y = 1.77 ± 0.20 kg, *F*_*1*,*48*_ = 4.44_,_ *p = 0*.*039*) were damaged (forebrain damage for lighter birds, brain stem damage in heavier birds).

## Discussion

This study provides a comprehensive assessment of the effects of three novel mechanical killing treatments (MARM, MZIN, and NMCD) and traditional manual cervical dislocation on laying hens and broilers at two ages. We captured iEEG signals and detailed behavioural/reflex responses, as well as post-mortem anatomical effects in the same individual birds, during application of on-farm dispatching methods. Manual cervical dislocation was the most reliable (100% success) followed by the NMCD (96%). This is the first assessment of true manual cervical dislocation [26] our findings suggest that welfare concerns raised previously may not be justified. However, since EU Regulation (1099/2009) limits MCD in birds weighing over 3 kg and the total number of birds to 70 birds per person per day, a reliable and humane mechanical alternative is required.

NMCD was easy to use and adaptable to different bird types and ages, although it was apparently limited by bird weight, as the three birds that weighed greater than 3.3 kg were not killed on the first attempt. This may have been due to the operator’s strength rather than the device itself and the same limitation may have applied to the MCD treatment had any of the birds in this group exceeded 3 kg (they did not). The reliability of NMCD concurs with reported high kill success rates of other mechanical cervical dislocation devices (e.g. Burdizzo and killing cone [3,11]).

The lower success rates observed when using MARM and MZIN devices were primarily due to difficultly in aiming the devices. The top heaviness of the MZIN as well as the small size of the bird’s head in relation to the bolt muzzle made it difficult to aim and balance the device on the head prior to firing. As the device was originally designed to kill rabbits, the significant difference in size (and ratio of head size to bolt muzzle) and shape of the skull of the target species meant that, despite the modifications made, the device was not reliable. Other captive bolt devices (e.g. pneumatically-operated nail gun–Draper Air Tools, UK; and Zephyr—NS 100A ¼ inch, Narrow Crown Stapler, Porter Cable, Jackson, TN) have been more successful, with 100% kill success in poultry [3,13].The MARM was difficult to apply to different sizes and types of birds. If a bird was slightly larger or smaller than the average bird for which the three individual head insertion cups had been designed [8], this resulted in the spike penetrating tissue in the wrong location and either limited or no brain damage occurring. The observed low kill success rate (< 50%), makes the MARM an unsuitable killing method for chickens.

The use of iEEG and behavioural recordings in this work to infer level of unconsciousness post device application had limitations, since the birds were anesthetized immediately prior to testing. However, we attempted to reduce ongoing anesthetic effects by using a rapid induction method which has shown in previous research to have minimal effect (i.e. rapid anesthetic recovery times) on the iEEG pattern [27–28] and reflex/behavioural durations [29]. Several studies have demonstrated that anesthesia alters iEEG patterns and derived spectral variables (e.g. PTOT and F50) [30]. Therefore from this study we cannot determine whether the killing treatments caused immediate loss of consciousness, since sevoflurane will have altered their brain state [27–28]. However, continuous analysis of spectral variables demonstrated that unconsciousness was maintained unconscious states after the 4 s anesthetic compromised interval until brain death.

Welfare impact was inferred in three ways: duration of reflexes (e.g. jaw/mandible tone), latencies to onset of particular iEEG frequency thresholds (e.g. F50 < 12.7 Hz; F50 < 6.8 Hz), and latencies to onset of previously validated unconsciousness thresholds. The baseline (awake) iEEG parameters concurred with the mean spectral variables reported in previous research [19]. As in previous work, unconsciousness was characterized by decreasing F50 and an increase in PTOT [18–19]. The F50 remained low after device application for all methods. The immediate increase of PTOT at the point of application has been suggested to indicate the loss of functional cerebro-cortical activity due to synchronization of firing neurons, increasing the overall amplitude [31]. The use of two unconsciousness thresholds was conservative and allowed a representation of changing brain state following application of each killing method. Interestingly, the latencies for both F50 thresholds were identical within killing method for NMCD and MARM, suggesting that when the method caused sufficient physiological trauma to result in loss of consciousness, it was immediate and resulted in a deep unconscious plane [19,32]. With MCD, the latencies for both thresholds (F50 < 12.7 Hz; F50 < 6.8 Hz) were different, possibly indicating a more gradual loss of consciousness.

The MCD method resulted in the shortest time to F50 < 12.7 Hz (2.6 s). However the mean latency to onset of permanent electrical brain activity below either threshold (F50 < 12.7 Hz; F50 < 6.8 Hz) was greater compared to first onset, demonstrating that the level of unconsciousness varied, but was below the sedation threshold [19] and was maintained until brain death. NMCD did not differ from MCD in terms of time to reach unconsciousness thresholds. Continuous sampling of iEEG and latencies to maintained unconsciousness thresholds (i.e. no epoch exceeded threshold post this time point) demonstrated that birds remained unconsciousness and well within the F50 < 12.7 Hz threshold post device application and until brain death. The MARM was the least humane with the longest durations to first onset for both F50 < 12.7 Hz and < 6.8 Hz, as well as longer latencies to permanent electrical brain activity below either threshold (F50 < 12.7 Hz; F50 < 6.8 Hz). However, it is important to note that around treatment application for all treatments (0–5 s) and at the onset of convulsions (e.g. severe wing flapping), recording clean iEEG was difficult due to potential high noise component in the trace from movement artefact [33]. This caused a reduction in usable epochs around these times.

Available iEEG data did provide an insight into the time to brain death (i.e. when the trace became isoelectric) for the MARM, NMCD and MCD. NMCD resulted in the shortest duration to first time isoelectric and last time not isoelectric compared to the only other mechanical device (MARM), though the difference between NMCD and MCD was not significant. The shorter duration to brain death may not have welfare benefits, but since operators must confirm the success of a kill immediately post-application and must not move on or kill another individual until the present one is confirmed dead (usually confirmed with lack of reflexes), the shorter the duration to brain death, the quicker these measures will cease and the operator can continue with their duties. This could indirectly benefit to bird welfare, as if operators are forced to wait for less time, they may be more likely to wait and confirm death, reducing the possibility that a severely injured bird would be left unattended for a prolonged period of time.

The duration of reflexes, the loss of which are considered to indicate death (e.g. nictitating membrane, pupillary and rhythmic breathing [5,12]) did not correlate with the derived duration to isoelectric signal from iEEG data. Both nictitating membrane and rhythmic breathing durations were considerably shorter for all killing treatments (all means < 10 s) compared to the mean durations to isoelectric (all > 20 s). Therefore, there is a risk that purely relying on these reflexes as an indication of death will incorrectly indicate that birds are dead before this is the case. The short durations of nictitating membrane persistence seen here do not agree with some previous research, in which mean durations for cervical dislocation methods ranged from 43–106 s, while for captive bolt methods all means were 0 s [3]. However, Martin et al [5], reported reflex durations for the MZIN, NMCD and MCD which are consistent with the results presented in this study [5]. The shorter durations observed here may be due to the physiological trauma being caused as a result of the killing methods. For example, damage to the brain stem (site of pupillary and nictitating membrane reflex control) can suppress blood supply to the eye [34], therefore affecting their responses. The short-acting sevoflurane anesthetic does not appear to have affected the durations adversely, since previously this anesthetic prolonged the cessation of such reflexes when birds were deeply anaesthetized [19].

Birds showed convulsive behaviors (e.g. leg paddling and wing flapping) post treatment application, and these behaviors continued beyond iEEG activity. This was true for all bird types, ages, and with all killing treatments. Similar results have been identified in previous research and confirm that the behaviors are not treatment specific [3,5,12,21]. Therefore these behaviors are not useful indicators of brain function and brain death, although their cessation could be used as a very conservative measure of brain death [3,21]. The last behaviour to cease in the majority of birds, irrespective of killing treatment, was cloacal movement and like other convulsive behaviors it continued for longer than any reflexes or electrical activity from the cerebral cortex. It has been suggested that the sporadic contraction and relaxation of the cloaca through spinal reflexes is not related to brain stem function and ceases once all available adenosine triphosphate (ATP) has been used within the muscle and sphincter [35].

As with reflexes and iEEG indicators of death, there was no correlation between reflexes and iEEG data for consciousness, highlighting a concern that established consciousness indicators for poultry as well as other species may not be accurate. However, the lack of relationship appears to be always in the same direction, with reflex durations being greater than the iEEG unconsciousness thresholds. When considering both measurements of reflex/behaviour durations and iEEG analysis (e.g. latencies and time series), the NMCD device appears to be the most humane mechanical method with no significant differences to MCD in terms of durations to unconsciousness. Despite the lack of iEEG data for the MZIN, the reflex data suggests it could be considered to be humane, as cranial reflex durations (pupillary, nictitating membrane, and rhythmic breathing), and jaw tone were abolished quickly post device application (all < 4.5 s). Therefore, if the reflexes are considered highly conservative compared to iEEG measurements, then hypothetically this treatment could be suggested to be the most humane, matching previous findings for captive bolt devices [3,11–12]. However, the rapid loss of reflexes must be taken within context of a relatively low kill success rate of only 75%, and the lack of reliability of this device means that it cannot be recommended for routine use in poultry.

For the two most promising methods, MCD and NMCD, all successfully killed birds had their cervical vertebrae dislocated and spinal cords severed. The location of the dislocation was very consistent, with approximately 80% of birds receiving a C0-C1 dislocation. This focusses the trauma around the brain stem and top of the spinal cord, resulting in functional impairment and increased likelihood of neurogenic shock [10]. The NMCD device was more consistent than MCD, causing severing/occlusion of the carotid arteries and maximizing cerebral ischemia and/or hypoxia [11].

Additional factors which at times affected the reliability, humaneness and consistency of all killing methods were bird type, bird weight and bird age. These were sometimes confounded resulting in interactions which could not be disentangled. The MARM and MZIN devices were most affected by these factors, demonstrating the limited ability of such devices to adapt to individual bird variation. The NMCD and MCD were minimally affected and were able to adapt to different sizes, weights and types of birds. This seems to be primarily due to the operator’s input into the application of the killing method, such that the methods could be subtly adjusted immediately (e.g. creating a wider gap between fingers, more vigorous stretch for larger birds, etc.), although this is reliant on the operator’s experience and training to make an assessment of what adjustments were required.

In conclusion, our evaluation of three mechanical killing devices and MCD with regard to their reliability, humaneness and consistency demonstrated that NMCD was the most successful novel method. The MZIN did show promise in terms of humaneness with the shortest reflex durations but this was countered by low reliability. The MARM device performed poorly in all three areas demonstrating its lack of suitability as a humane killing method for poultry on-farm. MCD method performed well as a killing method for poultry and matched the performance of NMCD. Collectively, the findings of this study provide evidence that the NMCD is a promising device for killing poultry on-farm, potentially fulfilling a need for a mechanical alternative to manual cervical dislocation.
